# Identification of key genes and their association with immune infiltration in adipose tissue of obese patients: a bioinformatic analysis

**DOI:** 10.1080/21623945.2022.2104512

**Published:** 2022-07-27

**Authors:** Jie Wen, Liwen Wang

**Affiliations:** a National Clinical Research Center for Metabolic Diseases, Metabolic Syndrome Research Center, Key Laboratory of Diabetes Immunology, Ministry of Education, and Department of Metabolism and Endocrinology, The Second Xiangya Hospital of Central South University, Changsha, Hunan, China; b Department of Endocrinology, Endocrinology Research Center, Xiangya Hospital, Central South University, Changsha, Hunan, China

**Keywords:** Obesity, adipose tissue, immune cell infiltration, gene expression omnibus, bioinformatic analysis

## Abstract

Immune cell-mediated adipose tissue (AT) inflammation contributes to obesity-related metabolic disorders, but the precise underlying mechanisms remain largely elusive. In this study, we used the R software to screen key differentially expressed genes (DEGs) in AT from lean and obese individuals and conducted function enrichment analysis. We then analysed their PPI network by using the STRING database. Hub genes were screened by cytohubba plugin. Subsequently, CIBERSORTx was used to predict the proportion of immune cells in AT from lean and obese subjects. Finally, the correlation between hub genes and immune cell proportions was analysed. These studies identified 290 DEGs in the AT between lean and obese subjects. Among them, *IL6, CCL19, CXCL8, CXCL12, CCL2, CCL3, CCL4, CXCL2, IL1B*, and *CXCL1* were proved to be hub genes in regulating the protein-protein interaction (PPI) network. We also found that *CXCL8* is positively correlated with resting NK cells, monocytes, activated mast cells, and eosinophils, but negatively correlated with CD8^+^ T cells and activated NK cells in obese individuals. Taken together, our study identified key genes in AT that are correlated with immune cell infiltration, uncovering potential new targets for the prevention and treatment of obesity and its related complications via regulating the immune microenvironment.

## Introduction

Obesity, which increases the risks for many metabolic dysfunctions including insulin resistance (IR) and type 2 diabetes mellitus (T2DM), has now become a global epidemic [1]. Accumulating evidence has revealed that obesity-induced low-grade chronic inflammation in AT plays a vital role in the progression of obesity and its related metabolic complications [2–4]. Numerous studies have shown that adipose-resident immune cells, such as T cells and macrophages, greatly contribute to adipose inflammation in obese humans and animals [5–8], which highlights the importance of maintaining the immune homoeostasis of AT and provides a potential therapeutic target for preventing obesity-associated diseases. However, the precise molecular mechanisms in recruiting the immune cells in AT in the context of obesity are still waiting for a full elucidation.

In this study, we used bioinformatic analysis to screen genetic alternation in the subcutaneous AT of obese individuals and identified several top-changed differentially expressed genes (DEGs) whose role in obesity is not clear. The Gene Ontology (GO) and the Kyoto Encyclopaedia of Genes and Genomes (KEGG) enrichment analyses of DEGs suggested the vital role of pathways including immune response and extracellular matrix (ECM) organization in the progression of obesity. We also screened several hub genes and analysed their correlation with immune cell proportions in AT of obese subjects. Our study revealed potential key genes and molecular mechanisms underlying obesity.

## Results

### Identification of 290 DEGs in adipose tissue between lean and obese individuals

**Figure 1.**
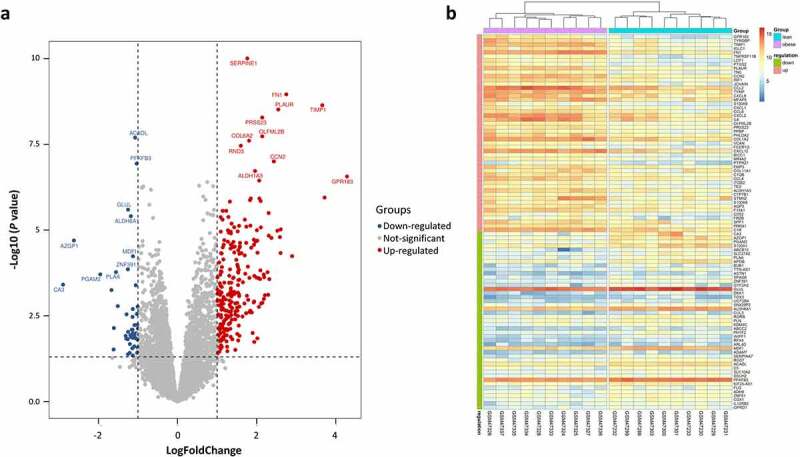
Differentially expressed genes (DEGs) between lean and obese individuals. (a) Volcano plot of all DEGs. Data points in red are up-regulated genes, and in blue are down-regulated genes. The top up-regulated and down-regulated genes are shown. (b) A Heatmap of the top 50 up-regulated DEGs and all down-regulated DEGs are shown.

After normalization of the microarray data from GSE2508, DEGs were screened based on defined criteria. A total of 290 DEGs including 245 up-regulated genes and 45 down-regulated genes were detected in obese individuals compared with lean individuals. The most significantly up-regulated and down-regulated genes were *GPR183* (logFC = 4.28) and CA3 (logFC = −2.89). *GPR183* is a newly identified gene that dramatically increased in AT of obese patients whose role in obesity is unknown. The lower expression of *CA3* in obese AT is consistent with previous findings [9]. Other top changed DEGs such as *SERPINE1, FN1, TIMP1, AZGP1*, and *ACADL* were shown in Figure 1(a), and the expression of the top 50 up-regulated DEGs and all down-regulated DEGs in each sample was visualized in Figure 1(b).

### DEGs are highly enriched in pathways involved in inflammation and extracellular matrix organization

**Figure 2.**
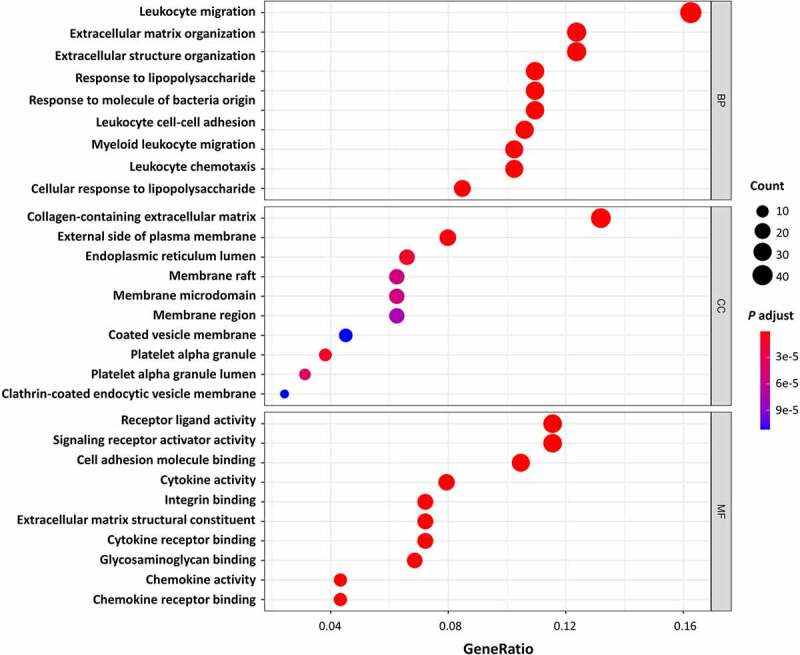
Gene Ontology (GO) enrichment analysis of all DEGs. The bubble shows the top 10 significant items according to the adjust *P*-value. BP, biological processes; CC, cellular components; MF, molecular functions.

**Figure 3.**
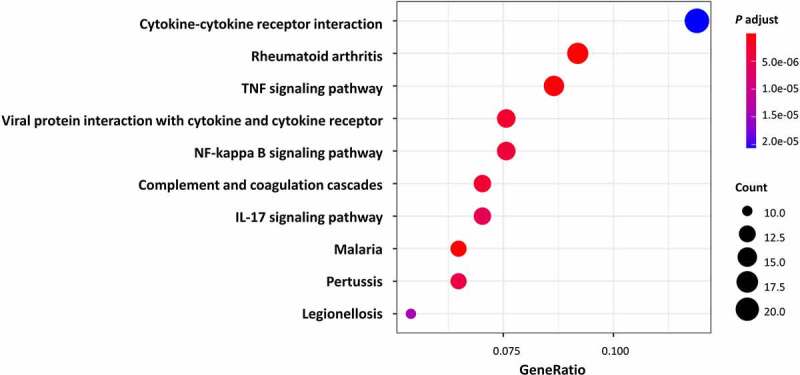
Kyoto Encyclopaedia of Genes and Genomes (KEGG) enrichment analysis of DEGs. Bubble shows the top 10 significant items according to the adjust *P*-value.

All DEGs were subjected to GO and KEGG pathway enrichment analysis. The results of GO enrichment indicated that for biological processes analysis, DEGs were mainly enriched in leukocyte migration, ECM organization, and extracellular structure organization (Figure 2 and Table 1). As for cellular components analysis, DEGs were enriched in the collagen-containing extracellular matrix, external side of the plasma membrane, and endoplasmic reticulum lumen (Figure 2 and Table 1). For molecular functions, DEGs were predominantly enriched in receptor-ligand activity, signalling receptor activator activity, and cell adhesion molecule binding (Figure 2 and Table 1). The results of KEGG pathway enrichment indicated that the DEGs were mainly enriched in cytokine-cytokine receptor interaction, IL-17 signalling pathway, TNF signalling pathway, and NF-kappa B signalling pathway, as well as pathways including Rheumatoid arthritis and some infective diseases, for instance, the Malaria, Pertussis, and Legionellosis. (Figure 3 and Table 2). Altogether, the DEGs of AT between lean and obese individuals are mostly enriched in the pathways associated with inflammation and ECM organization demonstrated by GO and KEGG analysis.

**Table 1.** Top Gene Ontology (GO) enrichment analysis of different genes expression.

Category	ID	Description	Count	Gene Ratio	*p*.adjust
Biologic process	GO:0050900	leukocyte migration	46	0.162544	1.59E-19
	GO:0097529	myeloid leukocyte migration	29	0.102473	2.36E-16
	GO:0030595	leukocyte chemotaxis	29	0.102473	9.69E-16
	GO:0030198	extracellular matrix organization	35	0.123675	3.09E-15
	GO:0043062	extracellular structure organization	35	0.123675	3.09E-15
Cellular component	GO:0062023	collagen-containing extracellular matrix	38	0.131944	2.13E-17
	GO:0009897	external side of plasma membrane	23	0.079861	3.33E-06
	GO:0031091	platelet alpha granule	11	0.038194	1.05E-05
	GO:0005788	endoplasmic reticulum lumen	19	0.065972	1.39E-05
	GO:0031093	platelet alpha granule lumen	9	0.03125	3.83E-05
	GO:0062023	collagen-containing extracellular matrix	38	0.131944	2.13E-17
Molecular function	GO:0005178	integrin binding	20	0.072202	9.86E-12
	GO:0005201	extracellular matrix structural constituent	20	0.072202	2.94E-10
	GO:0048018	receptor ligand activity	32	0.115523	7.12E-10
	GO:0005125	cytokine activity	22	0.079422	7.12E-10
	GO:0030546	signalling receptor activator activity	32	0.115523	7.48E-10

**Table 2.** Top KEGG enrichment analysis of different genes expression.

ID	Description	Count	Gene Ratio	*p*.adjust
hsa05323	Rheumatoid arthritis	17	0.092391	5.57E-09
hsa05144	Malaria	12	0.065217	9.96E-08
hsa04668	TNF signalling pathway	16	0.086957	3.29E-07
hsa04061	Viral protein interaction with cytokine and cytokine receptor	14	0.076087	2.63E-06
hsa04610	Complement and coagulation cascades	13	0.070652	2.63E-06
hsa04064	NF-kappa B signalling pathway	14	0.076087	3.49E-06
hsa05133	Pertussis	12	0.065217	4.34E-06
hsa04657	IL-17 signalling pathway	13	0.070652	5.60E-06
hsa05134	Legionellosis	10	0.054348	1.43E-05
hsa04060	Cytokine-cytokine receptor interaction	22	0.119565	2.13E-05

### PPI network construction, module analysis and hub genes selection

**Figure 4.**
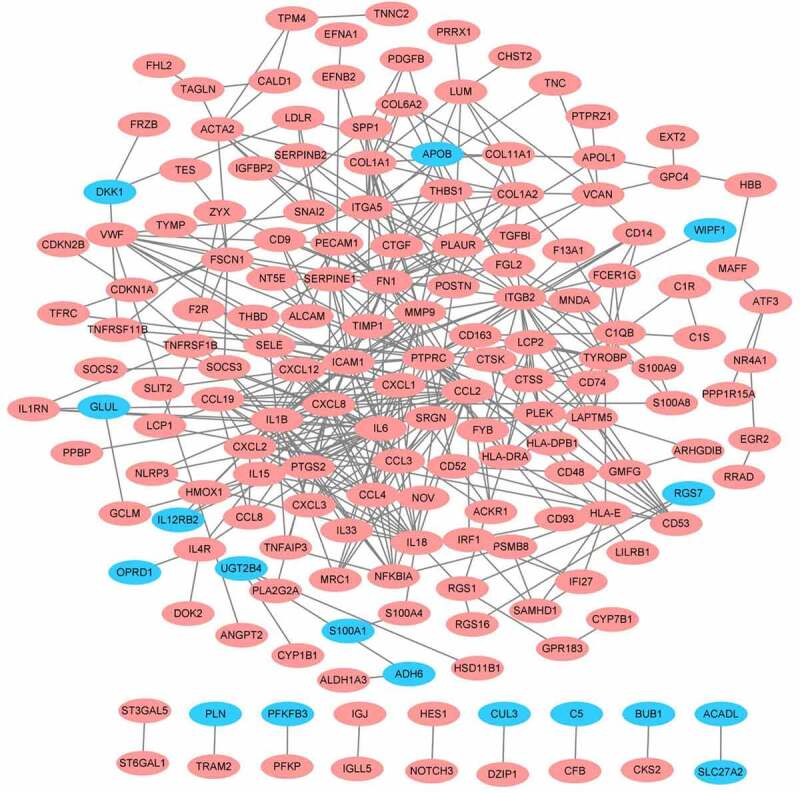
Protein-protein international network. Red represents up-regulated genes, blue represents down-regulated genes.

**Figure 5.**
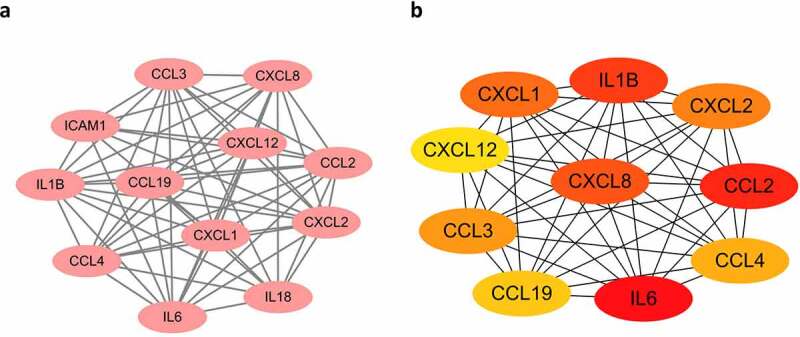
Modules and hub genes analysis. (a) Top 1 module of the PPI network is identified and visualized, all of them are up-regulated genes. (b) Top 10 hub genes of PPI network. Node colour reflects the degree of connectivity (Red colour represents a higher degree, and yellow colour represents a lower degree).

As shown in Figure 4, the PPI network of DEGs was generated and visualized in Cytoscape software based on the results obtained from the STRING database, which includes 169 nodes (genes) and 458 edges (interactions). The top significant module was identified by MOCEDE, which contains 12 nodes (MOCEDE score = 11.273, Figure 5(a)). The top 10 hub genes were selected by the MCC method in the Cytohubba plug-in, which are *IL6, CCL19, CXCL8, CXCL12, CCL2, CCL3, CCL4, CXCL2, IL1B*, and *CXCL1* (Figure 5(b). All of them are chemokines and cytokines, which highlight the immune dysfunction as a key point in obesity. Investigating the correlation between these hub genes and immune infiltration is necessary and requires further exploration.

### Analysis of immune cell proportion in adipose tissue

**Figure 6.**
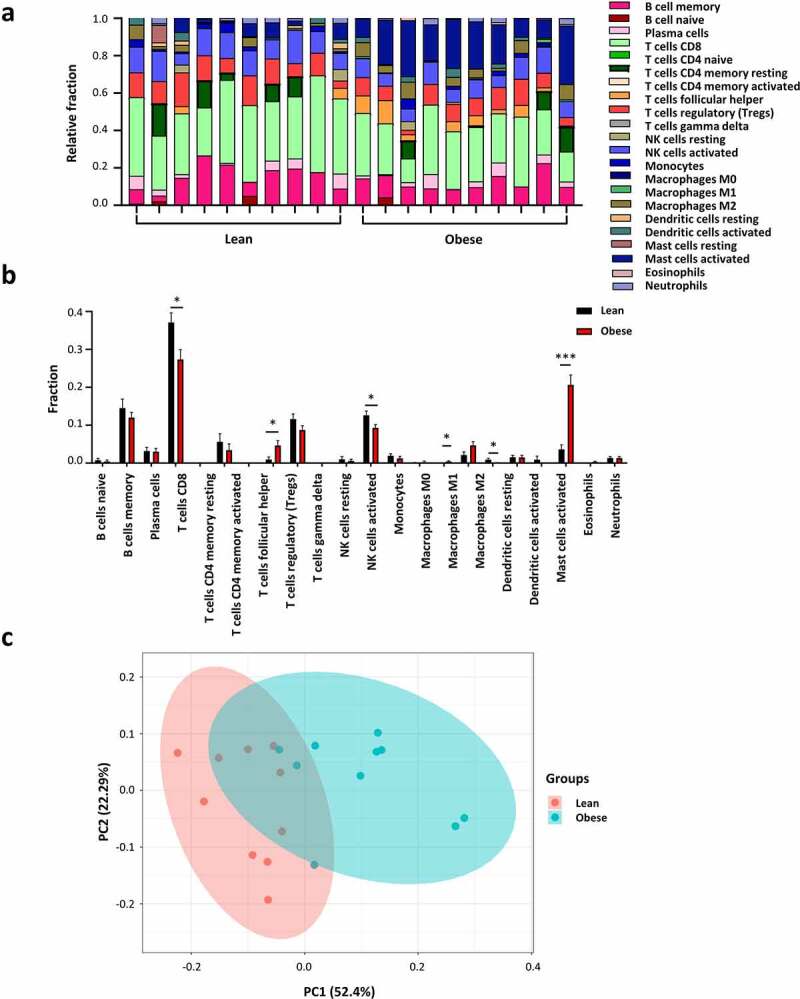
Immune cell proportions in adipose tissue from lean and obese individuals. (a) The stacked bar chart shows the proportion of immune cell populations in each sample. (b) The bar chart exhibits the difference of immune cell proportions in lean and obese subjects. (c) Principal component analysis (PCA) is performed on all samples. *p < 0.05, **p < 0.01, and ***p < 0.001.

Adipose depots show high cellular heterogeneity [10]. Over 15 populations of immune cells were identified in AT, and these immune cells participate in regulating the development and function of AT [11]. Investigating the changes in immune cell proportions or functions will be helpful in understanding the role of AT immunity in obesity. Then, we analyzed the difference in immune cell populations in AT from lean and obese subjects by the CIBERSORTx algorithm (Figure 6(a). To be noted, the 20 samples uploaded to CIBERSORTx did not match the generally accepted *P*-value <0.05. However, we consider the results drawn from CIBERSORTx to be reliable to some extent. Briefly, the quality of our data is high and PCA analysis exhibits distinct groups of integral gene expression (Fig. S1) and immune cell populations (Figure 6(c)). Additionally, the results from CIBERSORTx are consistent with those from other studies [8,12,13]. Obese individuals showed higher proportions of M1 macrophages, activated mast cells, and follicular helper CD4^+^ T (Tfh) cells. A negative correlation was observed between obesity and several cell types including CD8^+^ T cells, activated NK cells, and resting dendritic cells. However, our results showed no significant change in the proportion of M2 macrophages even though there is a trend of increase (Figure 6(b)), which could attribute to the small sample size and the differences within the group.

### The correlation analysis of hub genes with immune cell populations in adipose tissues of obese individuals

**Figure 7.**
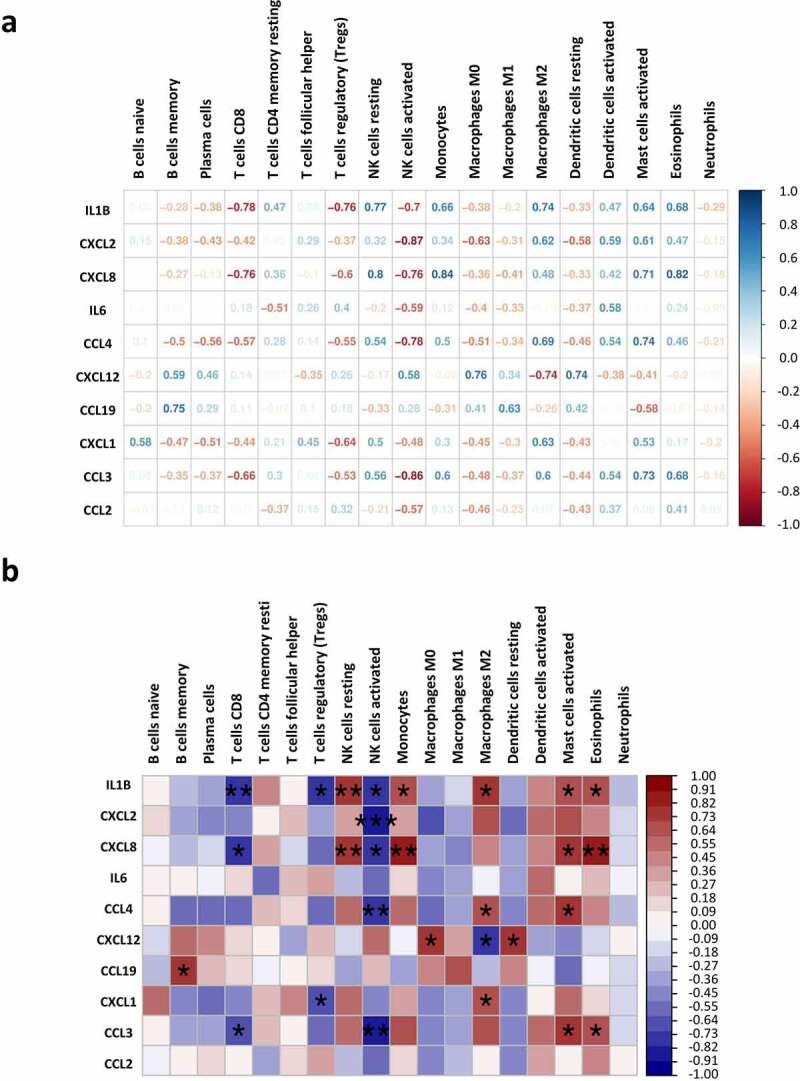
Correlation index between the expression of hub genes and proportions of immune cells in the obese group. (a) Numbers represent the correlation index. (b) Correlations represented by colour and statistic differences are shown. *p < 0.05, **p < 0.01, and ***p < 0.001.

All of the hub genes identified in Figure 5(b) are higher expressed in the AT of obese individuals. The association between these hub genes and the proportions of immune cells in AT were analysed by correlation analysis (Figure 7(a) and 7 (b)). We found that *IL1B* is negatively associated with the proportions of CD8^+^ T cells (r = −0.78, *p* < 0.01), regulatory T (Treg) cells (r = −0.76, *p* < 0.05) and activated NK cells (r = −0.7, *p* < 0.05), but positively related to resident NK cells (r = 0.77, *p* < 0.01), monocytes (r = 0.66, *p* < 0.05), M2 macrophages (r = 0.74, *p* < 0.05), activated mast cells (r = 0.64, *p* < 0.05) and eosinophils (r = 0.68, *p* < 0.05). *CXCL2* showed a negative correlation with activated NK cells (r = −0.87, *p* < 0.001).*CXCL8* is positively correlated with the proportions of resting NK cells (r = 0.8, *p* < 0.01), monocytes (r = 0.84, *p* < 0.01), activated mast cells (r = 0.71, *p* < 0.05), and eosinophils (r = 0.82, *p* < 0.01). *CCL4* showed positive correlation with M2 macrophage (r = 0.66, *p* < 0.05) and activated mast cells (r = 0.74, *p* < 0.05), but negatively correlated with activated NK cells (r = −0.78, *p* < 0.01). Moreover, *CXCL12* is positively correlated with M0 macrophage (r = 0.76, *p* < 0.05) and resting dendritic cells (r = 0.74, *p* < 0.05). The expression level of *CXCL1* is positively correlated with M2 macrophage (r = 0.63, *p* < 0.05) and negatively with Tregs (r = −0.64, *p* < 0.05). Another hub gene negatively associated with CD8^+^ T cells (r = −0.66, *p* < 0.05) and activated NK cells (r = −0.86, *p* < 0.01) is *CCL3*. The negative regulation effect of several hub genes in CD8^+^ T cells and activated NK cells may contribute to the lower proportions of them in obese AT as exhibited before (Figure 6(b)).

## Discussion

In this study, we identified and analysed 290 DEGs in AT between lean and obese subjects from the GSE2508 microarray dataset in GEO. As demonstrated by GO and KEGG enrichment analysis (Figure 2 and 3), these DEGs were mainly correlated to immune responses, ECM organization, and infective diseases. We further identified *IL6, CCL19, CXCL8, CXCL12, CCL2, CCL3, CCL4, CXCL2, IL1B*, and *CXCL1* as the hub genes that play vital roles in the pathogenesis of obesity. Moreover, our results suggested a negative correlation of *CXCL8* with CD8 + T cells and activated NK cells, and a positive correlation between them with resting NK cells, monocytes, activated mast cells, and eosinophils. In this paper, we uncovered several genes that may play central roles in regulating the immune infiltration in AT during the progression of obesity, which suggests possible novel targets for the future research or treatment of obesity in humans.

Although the correlations between most of the DEGs we identified in this study and obesity are well-established, such as *SERPINE1, PLAUR, CA3*, and *TIMP1* [9,14–16]. However, the function of other DEGs like *GPR183* and *PGAM2* in the progress of obesity is lacking and remains to be explored. *GPR183* encodes G protein-coupled receptor 183, which is one of the receptors of oxysterols and has been reported to be induced in the liver by high-fat diet feeding [17]. The well-known role of *GPR183* is to regulate lymphoid cell migration and activity [18,19]. However, no one revealed its function in AT. We proposed the possibility that upregulated *GPR183* may contribute to recruiting immune cells in AT under obesity conditions due to its crucial role in immune modulation. *PGAM2* encodes the key enzyme that participates in the glycolytic pathway and is highly expressed in heart tissue. Since the glycolytic beige fat has been emphasized in promoting energy metabolism and thermogenesis [20], regulating glycolysis in AT via targeting *PGAM2* could be a potential approach for obesity treatment. But all these possibilities need to be validated by future studies.

Enrichment analysis showed that significantly changed pathways are associated with immune response and ECM organization, for example, leukocyte migration, cell chemotaxis, extracellular structure organization, and ECM structural constituent. Our results confirmed the vital role of immunity and inflammation in the pathogenesis of obesity [21]. The dysregulation of immunity makes obese individuals suffer from persistent local and systematic low-grade inflammation. When these patients are faced with pathogens invasion, amounts of immune cells already existing in AT could trigger an excessive immune response and consequently induce the cytokine storm. These findings may partly explain why obesity is a risk factor for worse COVID-19 outcomes [22] and other infective diseases like malaria, pertussis, and legionellosis as shown in KEGG results (Figure 3). Meanwhile, we found ECM-related pathways occupies a large proportion in the results of GO analysis. During the obesity progression, AT undergoes dynamic remodelling which is composed of adipocyte expansion, immune cell accumulation, angiogenesis, collagen deposition, and fibrosis [23], most of which required ECM reorganization. Extra and abnormal deposition of ECM components may cause adipocyte necrosis and damage-associated molecular patterns (DAMPs) release, which will subsequently induce adipose inflammation and insulin resistance [23]. Hence, the regulation of ECM organization is essential for the treatment of chronic low-grade inflammation in obesity and its related complications.

AT is an energy-storage and endocrine organ, but also an immune organ [24]. A wide range of immune cells is involved in AT development and homoeostasis. Although CD8^+^ T cells exert important roles in the initiation and propagation of AT inflammation and remodelling during obesity [25], it is suggested that a high-fat diet reduces the total numbers and anti-tumour activities of CD8^+^ T cells in the tumour microenvironment [26]. Our study showed a reduced proportion of CD8^+^ T cells in AT from obese individuals compared to lean individuals, which may result from less CD8^+^ T cell accumulation in the late stage of obesity. Our results also showed more mast cells in the AT of obese subjects. It’s interesting given that mast cells have previously been shown to affect ECM remodelling and promote inflammatory cell recruitment and proliferation [27]. Meanwhile, accumulated mast cells in AT are regarded as critical contributors to obesity [28], which is in agreement with our findings. Tfh cells are important for germinal centre formation and maintenance [29]. Dysregulated Tfh cells in the intestine lead to microbiota dysbiosis and enhanced lipid absorption, which contribute to increased obesity [30]. No information is currently available on the role of Tfh cells in AT. We propose that, unlike Tfh in the intestine, Tfh cells may exert a detrimental role in AT homoeostasis. Further investigation would be needed to fully address this issue. Despite lower frequencies and reduced cytotoxic abilities of NK cells being observed in the blood of obese than in lean subjects [31], high-fat diet feeding increased NK cell numbers in epididymal AT and led to insulin resistance in animal models [32]. However, activated NK cells were downregulated in obesity in our result, suggesting a potential difference in NK cell regulation between humans and mice.

To explore the key factors that participate in regulating the immune microenvironment and deteriorating immune dysfunction in obese patients, we screened out the top 10 hub genes including *IL6, CCL19, CXCL8, CXCL12, CCL2, CCL3, CCL4, CXCL2, IL1B*, and *CXCL1* (Figure 5(b)) and analysed their correlation with immune infiltration in AT. Some of the hub genes have already been studied in obesity. And our results are consistent with these findings, proving high reliability for our subsequent correlation analysis. All of the hub genes are significantly upregulated in the AT of obese subjects, and several of them were top changed DEGs as shown in the heatmap (Figure 1(b)). The most significant hub gene identified in our study was *IL6*, which exerts pleiotropic roles in inflammation and metabolic diseases [33]. Previous studies suggest that IL-6 is a proinflammatory cytokine that plays an important role in white adipose tissue dysfunction and hepatic insulin resistance [12,34], which was consistent with our results. But we failed in linking *IL6* with immune infiltration due to its major role in aggravating inflammation but not attracting cells. Other chemokines, for instance, *IL1B, CXCL8, CCL4, CXCL12, CCL19, CXCL1, and CCL3* exhibited positive or negative correlations with the proportions of some immune cells. *CXCL8* expression is upregulated in AT of obese individuals, which induces chemotaxis of neutrophils and other granulocytes to AT [13,35]. CXCL12 could promote cancer progression and is a potent chemoattractant for haematopoietic cells [36].

CXCL8 is largely produced by immature noncytolytic NK cells and regulates early NK cell differentiation [37]. Tumour-infiltrating monocytes also secret CXCL8, which is positively associated with human hepatocellular carcinoma [38]. In addition, CXCL8 produced by circulating eosinophils also contributes to the airway inflammatory responses within allergic lesions [39]. However, to our knowledge, the roles of CXCL8 within NK cells, monocytes, and eosinophils in the progression of obesity have not yet been identified. Our results propose that CXCL8 may be a potential target of obesity in these cells. CXCL12 has been reported as an adipokine that recruits macrophages to AT and induces obesity-related inflammation and systematic insulin resistance [40]. The proinflammatory property of CXCL12 is inversely correlated with M2 macrophages (Figure 7).

This study also has some limitations. Firstly, the sample size was relatively small, although part of the results could be validated by others’ reports. Secondly, the parameters of specific individuals were not offered. Lastly, our results still need future experiments to uncover the role of these genes in the pathogenesis of obesity and to explore their possibilities as therapeutic targets for obesity. Nevertheless, we comprehensively studied the correlation between gene expression and immune cell populations in AT of lean and obese individuals. Through this bioinformatic analysis, we identified several unreported genes that are closely related to obesity and revealed unrecognized molecular mechanisms in immune cells infiltration during obesity, which may provide potential cellular and molecular targets to be explored for their roles in the progression of obesity.

## Materials and methods

### Microarray data

We downloaded the gene expression dataset GSE2508, which contains a total of 20 human white AT samples from lean (BMI≤30, n = 10) and obese (BMI>30, n = 10) individuals [41], from the Gene Expression Omnibus (GEO) database (https://www.ncbi.nlm.nih.gov/geo/), a public functional genomic data repository. The microarray data of the GSE2508 dataset was identified by GPL8300 platforms (HG_U95Av2; Affymetrix Human Genome U95 Version 2 Array).

### Identification of differentially expressed genes

After annotating the dataset with the R script in R 4.0.0 software (https://www.r-project.org/), we used the *‘limma’* V3.44.3 package to screen DEGs in AT between lean and obese individuals [42]. The data were first normalized using the *‘normalize Between Arrays’* function from *‘limma’* (Fig. S2). The t-test and eBayes methods were then used to calculate the fold change (logFC) and the *P*-value [43]. Lastly, significantly changed DEGs were identified using the cut-off thresholds of |logFC| >1 and p < 0.05. The *‘pheatmap’* V1.0.12 package was used to draw a heatmap of top 50 up-regulated and all down-regulated DEGs in R software. The volcano of DEGs was visualized by *‘ggplot2’* V3.3.2 package.

### Function and pathway analysis of DEGs

GO analysis offers a model that categorizes gene function into three parts: biological processes, cellular components, and molecular functions [44]. The KEGG is a database that provides a comprehensive set of bio-interpretation of genomic sequences and protein interaction network information [45]. In our study, GO and KEGG analyses of DEGs were completed by *‘clusterProfiler’* V3.16.0 package [46].

### Construction of protein-protein interaction (PPI) network, module analysis and identification of hub genes

The Search Tool for the Retrieval of Interacting Genes (STRING 11.0; https://string-db.org/) is an online tool to predict the interactions of genes at the protein level, including direct (physical) and indirect (functional) interactions [47]. The PPI network of DEGs was constructed with a high confidence > 0.7. Subsequently, the PPI network was visualized in Cytoscape software 3.8.0 (https://cytoscape.org/) [48]. The significant modules of the PPI network were selected by Molecular Complex Detection (MCODE) V1.6.1 plug-in with the default parameters (degree cut-off, 2; K-Core, 2; max depth, 100; node score cut-off, 0.2) [49]. Moreover, cytohubba, another plug-in in Cytoscape, was used to explore important nodes with 11 methods (the MCC method is widely used and meets a satisfying comparative performance), subsequently for studying hub genes [50].

### Immune cells proportion analysis

CIBERSORTx is an online analytical tool that estimates the relative levels of 22 phenotypes of human haematopoietic cells in a mixed cell population using gene expression data [51]. Gene expression data was uploaded to CIBERSORTx (https://cibersortx.stanford.edu/), then the algorithm was run using signature matrix LM22 provided by CIBERSORTx and 500 permutations. Principal component analysis (PCA) was performed by *‘ggplot2’* V3.3.2 package to determine whether different immune cell populations existed between the two groups.

## Supplementary Material

Supplemental Material

## Data Availability

The datasets generated and/or analyzed during the current study are available in the GEO database (https://www.ncbi.nlm.nih.gov/geo/query/acc.cgi?acc=GSE2508). Data are available upon request to the corresponding author.

## Statistical analysis

A two-tailed Student’s t-test was used to evaluate the differences in immune cell proportions in AT between lean and obese individuals. The correlation between the expression of hub genes and the population of immune cells in obese subjects was calculated by Pearson correlation analysis.
