# Supplementary material for: Identification of key genes and their association with immune infiltration in adipose tissue of obese patients: a bioinformatic analysis
Source: Adipocyte. 2022 Jul 27;11(1):401–12. doi: 10.1080/21623945.2022.2104512 (PMC9336476; doi:10.1080/21623945.2022.2104512)
Supplement: Supplemental Material [file KADI_A_2104512_SM4755.pdf]

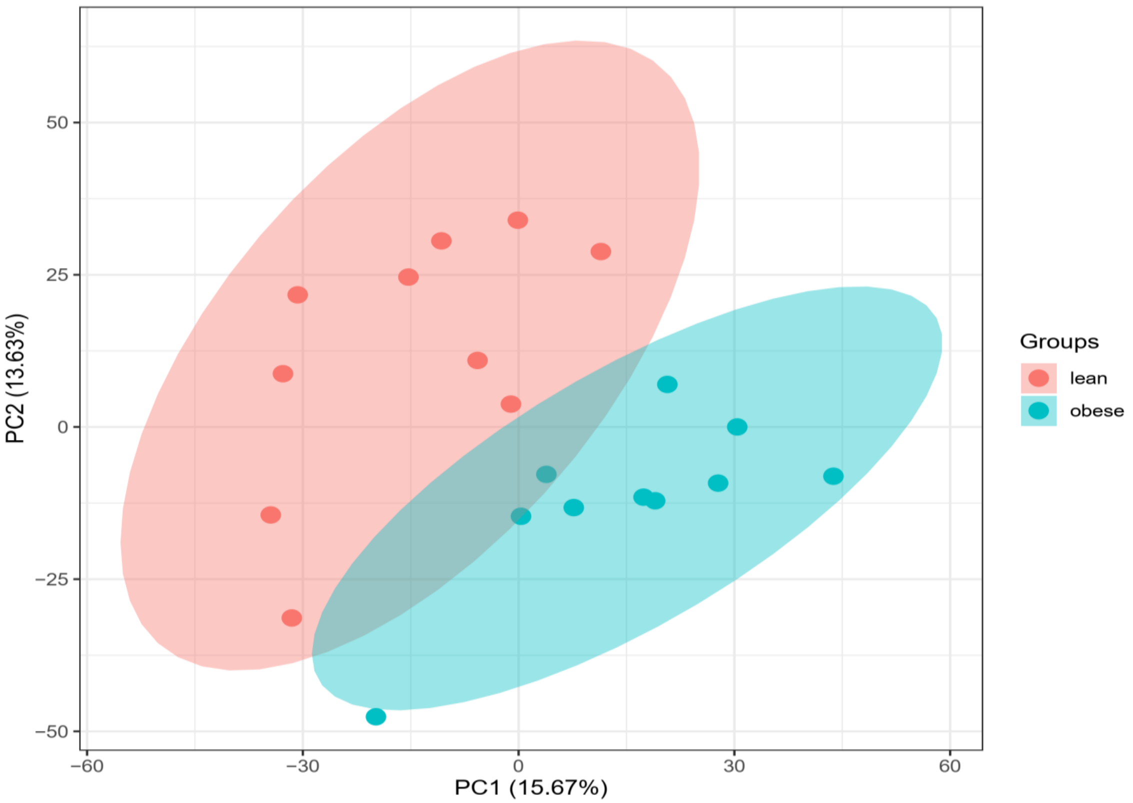


**Figure S1. Quality of selected dataset.** Principal component analysis performed gene expression on each sample.

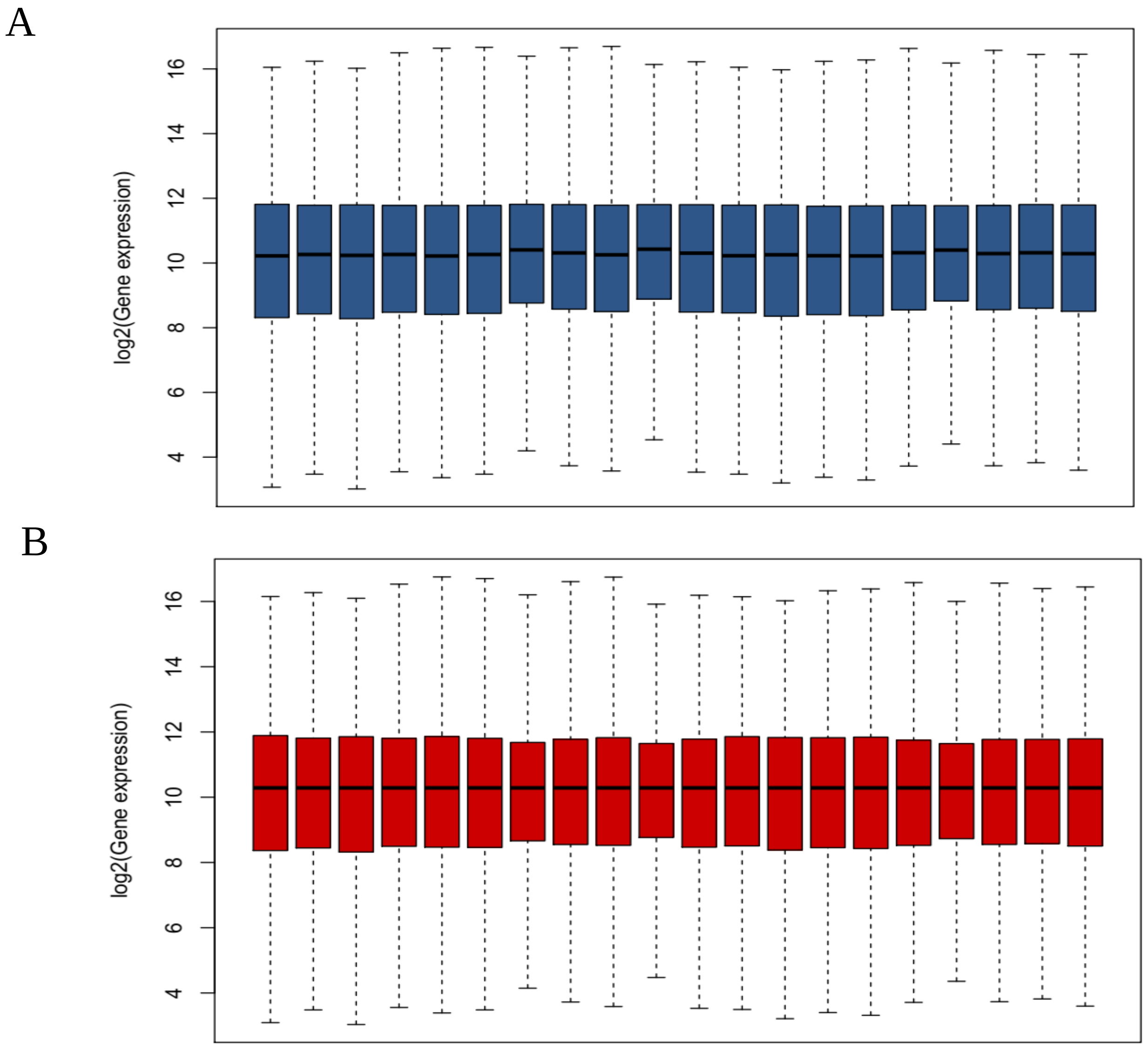


**Figure S2. Box plot of gene expression data before and after normalization.** (A) The blue bar represents the data before normalization; (B) The red bar represents the data after normalization.
